# Dr. Buel Goodsell Streeter

**Published:** 1900-09

**Authors:** 


### OBITUARY.

Dr. Buel Goodsell Streeter, of Glens Falls, N.Y., died at his home in that city July 24, 1900, aged 68 years. He entered the volunteer service during the civil war as assistant surgeon of the Ninth New York Cavalry, was promoted to surgeon of the Fourth New York Cavalry, May 29, 1863, resigned September 24, 1863, re-entered the service as surgeon U. S. Volunteers and became medical director of the army of the Shenandoah. After he was mustered out of service he returned to Glens Falls, where he entered upon the active practice of medicine, and thus continued until a short time before his death.

Dr. Streeter was one of the first citizens of the region of his residence, served prominently as a member of various medical societies, took active interest in educational matters and was for many years chairman of the local school board. He was for a long period president of the board of pension examining surgeons at Glens Falls and a companion of the Military Order of the Loyal Legion. He died respected by the entire community and his funeral was very largely attended. His widow, a son, Dr. F. B. Streeter, and two daughters survive.

